# Development of Automated Visual Acuity Measurement Using a Calibration-Free Eye-Tracking System

**DOI:** 10.7759/cureus.64401

**Published:** 2024-07-12

**Authors:** Noriaki Murata, Haruo Toda, Hokuto Ubukata, Mao Takagi, Chie Tanaka, Ai Machinaga, Minami Miyajima, Shunya Tatara

**Affiliations:** 1 Department of Orthoptics and Visual Sciences, Niigata University of Health and Welfare, Niigata, JPN

**Keywords:** monocular vision, grating acuity, visual acuity testing, vision screening, eye-tracking technology

## Abstract

Purpose

Infant vision assessment often relies on grating acuity; however, its objectivity and convenience must be improved. A calibration-free eye-tracking system, even in preverbal children, enables easy and precise gaze analysis. This pilot study aimed to develop a reliable automated monocular vision screening.

Methods

Participants (n=118) underwent a grating visual acuity test using the eye-tracking system. Correlations between the grating acuity, uncorrected visual acuity, and refractive error were analyzed across different cutoff values of fixation duration percentage.

Results

Strong correlations were found between the grating acuity and refractive error at 69% and 88% thresholds. Similar correlations with uncorrected visual acuity were noted at 70% and 89% thresholds. False-negatives around the 70% threshold were noted, indicating potential overestimation of acuity in cases of low visual acuity/high refractive error.

Discussion

The results highlight the feasibility of calibration-free eye-tracking system-based monocular vision screening with an optimal screening threshold of 90%.

## Introduction

Various factors can hamper the normal development of infantile vision. For instance, untreated amblyopia resulting from strabismus, severe refractive error, or vision deprivation-induced poor retinal image quality during the visual critical period in childhood may lead to compromised vision in adulthood [1]. Thus, early detection and intervention for amblyopia are crucial because individuals aged ≥8 years are considered less responsive to treatment; accordingly, it must be identified early to secure their future visual acuity [2].

In 2021, the American Association for Pediatric Ophthalmology and Strabismus (AAPOS) established comprehensive guidelines for device-based childhood vision screening [3]. These guidelines specify criteria, including refraction, turbidity of the intermediate optic media, and strabismus, for identifying amblyopia-associated risk factors. However, assessment methods of visual acuity must be improved to aid in the early detection of amblyopia-related visual impairments. A device that can rapidly estimate visual acuity levels in infants, when used in conjunction with refraction tests, may reduce the likelihood of overlooking amblyopia.

Decimal visual acuity is widely used in Japan. It is evaluated using a Landolt chart (shaped like a C) placed 5 m from the patient. The visual-spatial resolution, that is, the ability to distinguish two closely located points or lines is evaluated based on the subjective response. That is, the patient must decide the position of the gap (right, top, left, or down) in the Landolt chart. Decimal visual acuity is expressed as the reciprocal of the minimum angle of resolution (MAR) in minutes, which is formed by two straight lines from the ends of the object to the nodal point of the eye. In healthy adults, the subjective refraction test, a psychophysical method quantifying visual acuity through subjective responses, is often employed in vision screening.

On the contrary, grating visual acuity is used for preverbal infants. Infants characteristically prefer looking at grating patterns over homogeneous surfaces, and this is used to examine whether the examiner, such as a certified orthoptist, is observing the eye movements of the infants and looking at the patterns [4,5]. By determining this, it may be converted into a visual acuity value, even without a subjective response. Theoretically, MAR (i.e., visual acuity) can be measured using Landolt charts and grating patterns with various spatial frequencies. In infants, vision screening relies on acuity card procedures, such as the teller acuity card (TAC) technique, based on preferential looking, which is considered the gold standard [6-8].

Although the grating acuity card technique is deemed reliable for monocular vision screening, this method involves two overlapping psychological processes [9]. In this technique, not only the tested infant but also the examiner must make a judgment because the examiner identifies the eye gaze without knowing the side of the card presenting the grating pattern [8].

Eye tracking technology has emerged recently, and ophthalmic studies have explored its application to visual function tests, including eye movements, objective visual field, and contrast sensitivity [10-13]. Some studies have also investigated the use of an eye tracker in grating acuity testing for vision screening [14-18]. Vision screening using an eye tracker eliminates examiner subjectivity and is unaffected by their skill level, and this method may help improve vision screening in infants who have not yet acquired spoken language.

However, no gold standard eye-tracking system has been established for grating acuity testing, and existing systems employ different test parameters. Therefore, experiments with various eye-tracking systems prove beneficial. However, eye-tracking systems need to calibrate the gaze direction before screening. Thus, in this study, we aimed to perform grating acuity testing using a calibration-free eye-tracking system to identify test parameters suitable for general use. The proposed eye-tracking system features an infrared facial recognition function, eliminating the need for calibration and initiating eye tracking by positioning the examinee in front of the system. This pilot study explored the correlation between grating acuity calculated using a calibration-free eye-tracking system and refractive error and visual acuity was measured using Landolt charts in healthy adults.

## Materials and methods

Participants

This study was approved by the ethics committee of the Niigata University of Health and Welfare (Approval no. 18627-210528) and adhered to the principles outlined in the Declaration of Helsinki. Data were collected from May 31, 2021, to June 30, 2023. The participants provided written informed consent for the publication of case details. All data without participant identifiers were transferred to a secure university computer. The study included 123 participants (123 left eyes) without ophthalmic conditions, except for refractive errors. Before enrollment, candidates were subjected to a 5 m Landolt visual acuity test to confirm a logarithm of the MAR (logMAR), corrected visual acuity of ±0.0. They also underwent Humphrey Field Analyzer (Carl Zeiss Meditec, Inc., Dublin, CA, USA) 24-2 Swedish Interactive Threshold Algorithm-standard visual field testing and the non-mydriatic fundus camera nonmyd WX (KOWA, Tokyo, Japan). The study excluded those who had corrected visual acuity deterioration, met the glaucomatous visual field criteria by Anderson and Patella, or had abnormalities in the posterior segment [19]. Spherical equivalents were calculated based on objective refraction measurements obtained using an ARK-1a automatic refractometer (Nidek Co. Ltd., Aichi, Japan). Uncorrected visual acuity was measured using an 80 cm Landolt chart specifically created for this study by a precise printing provider, aligning with the maximum test distance of the eye-tracking system detailed below. According to the AAPOS criteria, we used objective refraction values. Moreover, we collected data on the Landolt chart, which shows visual acuity at 80 cm and allows for a comparison directory because young adults may have become myopic due to the inclusion of instrument myopia.

Calibration-free eye-tracking system

The EMR ACTUS (Nac Image Technology, Tokyo, Japan) eye-tracking system is equipped with a screen and utilizes the bright pupil technique for eye tracking. An infrared facial recognition function initiates eye tracking once the examinee is positioned in front of the system. After facial recognition, monocular eye tracking can be sequentially performed with one eye covered at a time. The system incorporates CALFREE technology to estimate the optical axis when at least two cameras and two-point light sources are available [20]. The sampling rate, spatial resolution, and accuracy were 50 Hz, 2.0°, and 4.0° in calibration-free mode, respectively. The display resolution was 1920 × 1080 pixels.

Grating acuity experiment with the EMR ACTUS system

**Figure 1:**
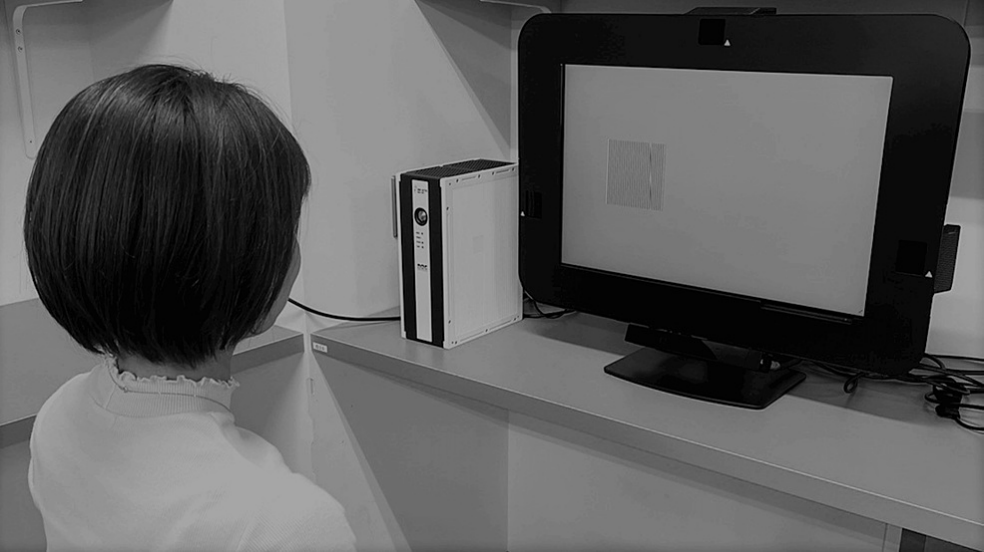
EMR ACTUS setup during the grating acuity experiment The facial recognition function allowed for calibration-free eye tracking. This started after an examinee sat in front of the system without a mask or sunglasses.

**Figure 2:**
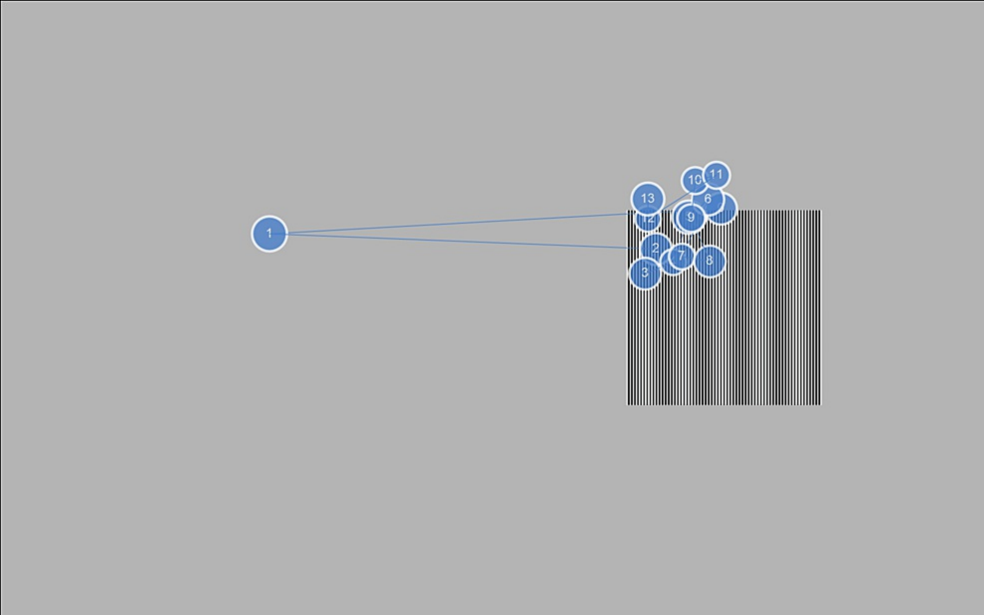
Eye movements during the grating acuity experiment Circles indicate fixation and lines indicate saccades. The number in the circle is the rank of fixation. Immediately after the measurement starts, the gaze is at the center of the screen; however, once the grating pattern is recognized, the gaze converges on it. This allows us to visualize that the grating pattern is visible.

With uncorrected refractive errors, participants focused on grating patterns simulating TACs displayed on the EMR ACTUS system screen. No chin support was used (Figure 1). Because visual acuity tends to be better when the examination distance is shortened, the examiner carefully monitored the distance. In addition, EMR ACTUS has a maximum examination distance of 80 cm. If the examination eye is located at a distance greater than this, measurement is impossible, so the reproducibility of the distance was maintained. This system performed binocular visual acuity measurements in the following sequence: first, a slide was presented with instructions to gaze at a grating pattern, during which the subject's gaze was calibrated using face recognition; second, a slide was presented with instructions to cover the left eye, and then the right eye was measured; finally, a slide was presented with instructions to cover the right eye, and then the left eye was measured. The brightness of the gray background was set to 180 based on the results of the flicker method in three observers. This is applicable only to the LCD monitor that comes with the device. The appropriate background brightness depends on the gamma value of the display used for the target presentation. If the background brightness was not appropriate, it would cause an underestimation of the results (even participants with low visual acuity could choose the correct panel using the brightness cue rather than the stripe). The height and width of the grating patterns on the screen were 10 cm and 10 cm, respectively. They were positioned at an inner visual angle of 10° and an outer visual angle of 25° on the left or right side of the screen, and 1-, 2-, or 3-pixel gratings created patterns with spatial frequencies of 25.8, 12.9, and 8.4 cpd, respectively, for measuring grating acuity at a test distance of 80 cm. For each spatial frequency, three grating patterns were displayed for 4 s each. The mean percentage of the participant’s fixation duration on the left or right half containing the contacting pattern was estimated for each spatial frequency (i.e., a mean of three patterns). During the examination, eye movements can also be observed and recorded using the visualization function (Figure 2).

Data analysis

**Figure 3:**
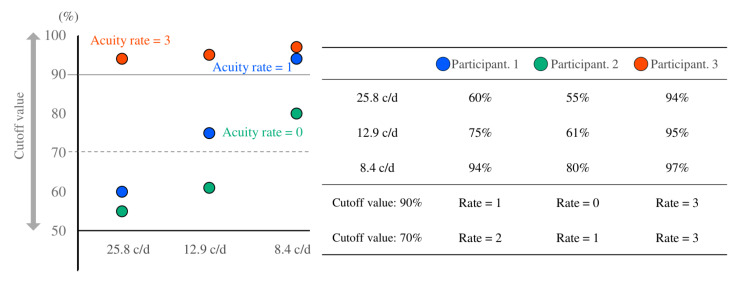
Determination of the grating visual acuity rating The participant’s visual acuity rate varies by the cutoff value based on the percentage of fixation duration. The cutoff value was between 50% and 99%. The visual acuity rate shown was obtained with a cutoff value of 90%. We confirmed the relationship among refractive error, uncorrected visual acuity with the Landolt chart, and grating visual acuity rating obtained through this method.

The grating visual acuity rating was determined based on the fixation duration in the presentation time of the grating pattern. Based on the relationship between the grating visual acuity rate and conventional indicators, the cutoff of the mean percentage of fixation that determines the grating visual acuity rating was examined. Ratings were as follows: visual acuities of 25.8, 12.9, 8.4, and <8.4 cpd were rated 3, 2, 1, and 0, respectively. Cutoff values were between 50% and 99%. For instance, if the mean fixation duration rates on a grating pattern in three attempts for 25.8, 12.9, and 8.4 cpd were 60%, 75%, and 94%, respectively, and the cutoff was 90%, the participant was determined to visualize a grating pattern of 8.4 cpd, resulting in a rating of 1. The visual acuity rating determination using three simulated participants is presented in Figure 3. Using the Spearman rank-correlation coefficient (ρ), the correlation between visual acuity level and spherical equivalent based on objective refraction testing was examined, as well as the uncorrected visual acuity measured using an 80 cm Landolt chart at cutoff values of 50%-99%.

This study enrolled young adults with a best-corrected visual acuity of 20/20 or better and participants with far points ≥80 cm (myopia <1.25 D); theoretically, the grating acuities of the participants were expected to be rated 3. Therefore, ρ was estimated for all participants and the subset with myopia >1.25 D. Statistical analyses were performed using IBM SPSS Statistics for Windows version 23 (IBM Corp., Armonk, NY, USA), with a significance level of 5%.

## Results

Data could not be collected from five (4.2%) participants because of tracking errors and errors in face recognition when one eye was covered. The analysis included data from 118 participants (mean age, 20.0 years; standard deviation, 1.1 years). Among the participants, the mean objective refractive error was −3.71±2.89 D (range, +4.88-−13.75 D), and the mean 80 cm Landolt visual acuity was +0.5±0.4 (range, 0.0-+1.1) logMAR.

Overall participant analysis

**Figure 4:**
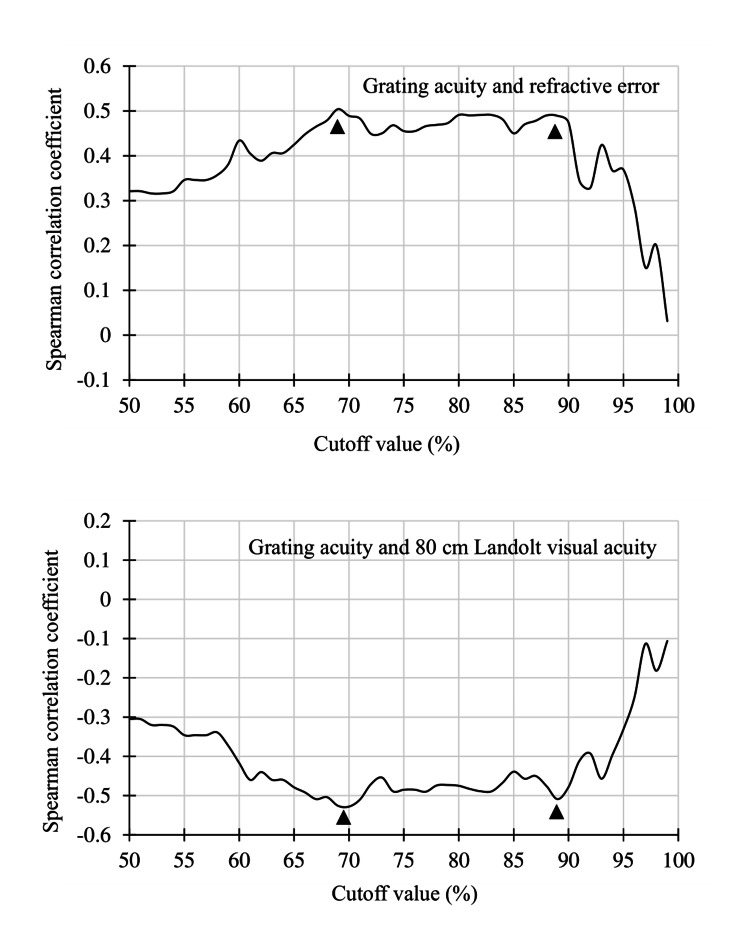
Spearman correlation coefficients at 50%-99% of the cutoff value among all participants A bimodal distribution was observed between the grating acuity and 80 cm Landolt visual acuity at 70% and 90% cutoff (arrowheads). The correlation coefficient was lower at thresholds >90%.

Among all participants (n=118), a significant correlation was found between the objective refractive error and the grating acuity level and between the 80 cm Landolt visual acuity and the grating acuity rate within the cutoff values of 50%-96% (P<0.01). For these participants, the objective refractive error strongly correlated with the grating acuity rate at cutoff values of 69% (ρ=0.50) and 88% (ρ=0.49). A strong correlation was also observed between the 80 cm Landolt visual acuity and the grating acuity rate at cutoff values of 70% (ρ=−0.53) and 89% (ρ=−0.51) (Figure 4).

Analysis of participants with myopia ≥1.25 D

**Figure 5:**
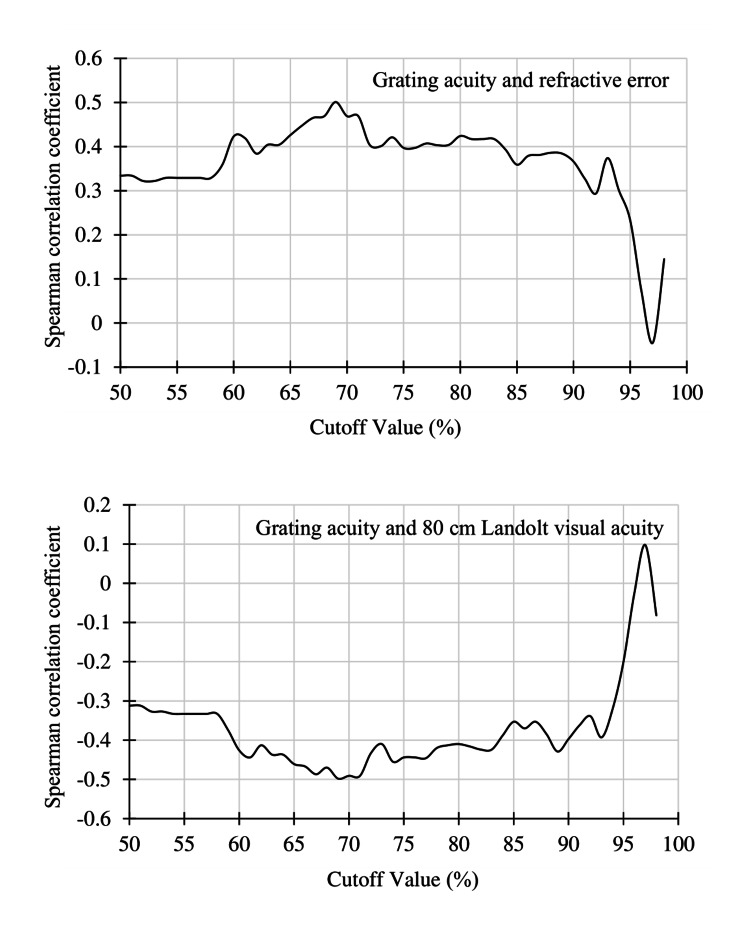
Spearman correlation coefficients at 50%-99% of the cutoff among participants with myopia >1.25 D Compared with the plots for all participants, the highest correlation occurred around the cutoff value of 70%.

In the analysis of participants with myopia ≥1.25 D (n=96), a significant correlation was found between the objective refractive error and the grating acuity rate and between the 80 cm Landolt visual acuity and the grating acuity rate within cutoff values of 50%-95% (P<0.01). Among these participants, a strong correlation was found between the objective refractive error and the grating acuity rate at cutoff values of 69% (ρ=0.50) and 88% (ρ=0.39) and between the 80 cm Landolt visual acuity and the grating acuity rate at cutoff values of 69% (ρ=−0.50) and 89% (ρ=−0.43). The strongest correlation was found at the cutoff value of 70% (Figure 5).

Distribution of uncorrected visual acuity measured using a Landolt chart of participants with a grating acuity rate

**Figure 6:**
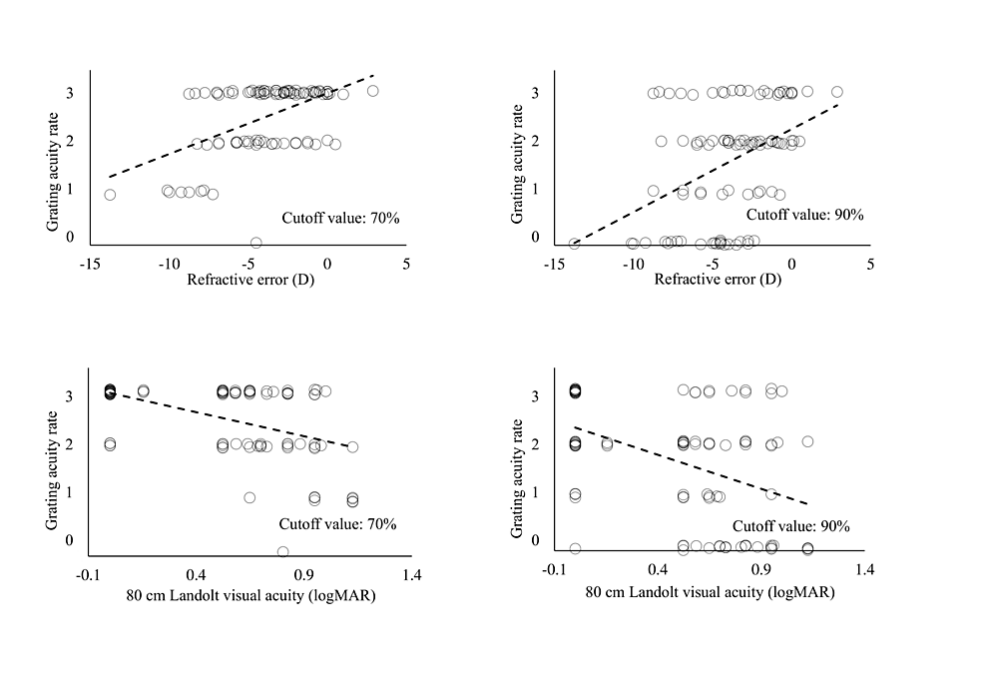
Scatter plots for all participants at cutoff values of 70% and 90% The estimated absolute correlation coefficients showed significant correlations at cutoff values of 70% and 90%. However, in the diagram for 70%, values were concentrated at grating acuity level 4, the best visual acuity classification. Therefore, a high false-negative rate (proportion of participants falsely determined to have good vision) was suspected. logMAR: logarithm of the minimum angle of resolution

**Figure 7:**
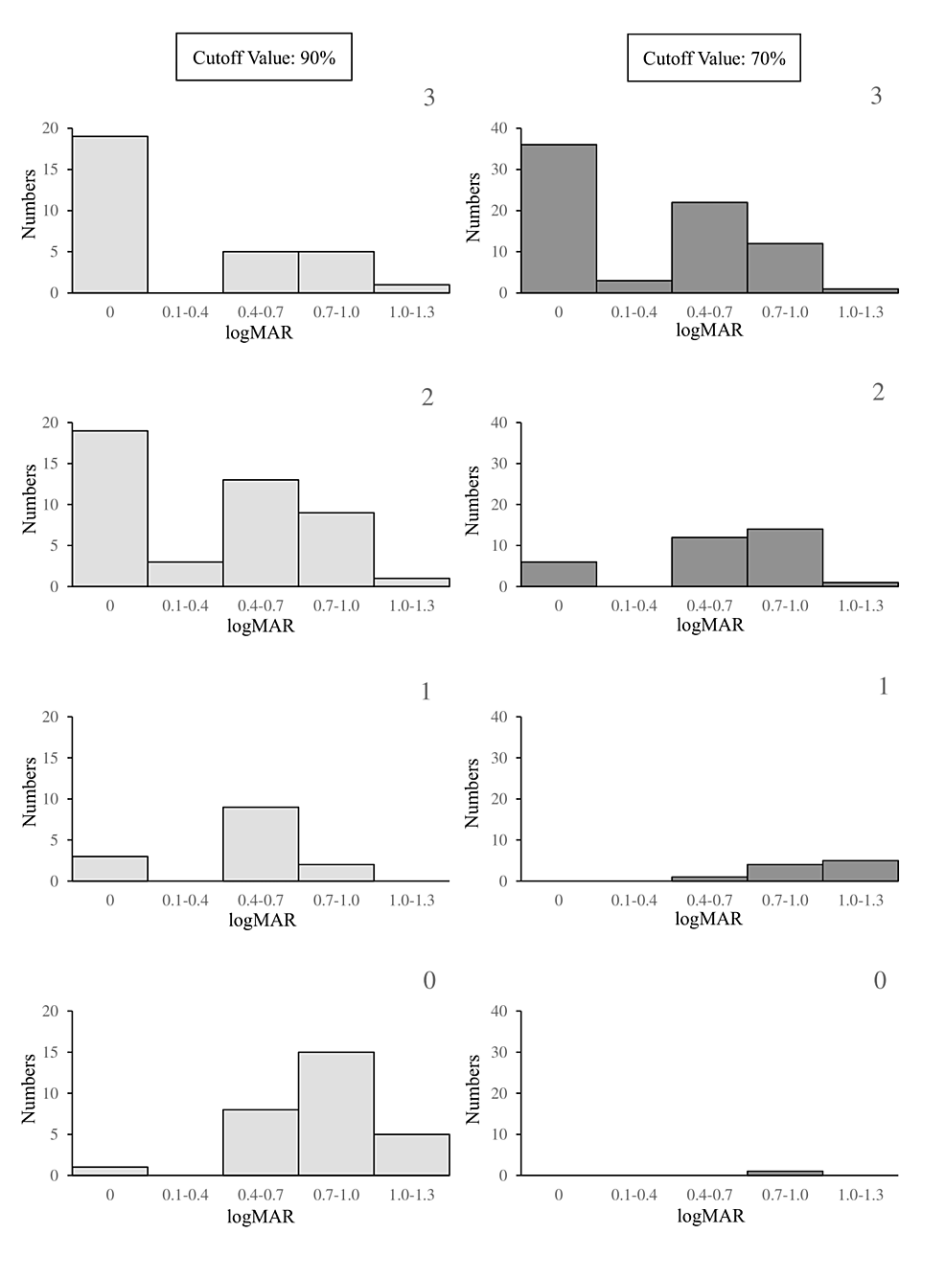
Histograms of uncorrected visual acuity measured using the Landolt chart of participants by the grating acuity level At a cutoff value of 70%, even participants with significant visual acuity decline were classified in grating acuity level 4 at a high rate, suggesting they were unsuitable for screening. logMAR: logarithm of the minimum angle of resolution

Given the good correlation between the grating acuity level and the refractive error or Landolt chart visual acuity at 70% and 90% thresholds, scatter plots were observed for all participants at 70% and 90%. Figure 6 illustrates the correlation among the grating acuity rate, 80 cm Landolt chart visual acuity, and refractive error. Many plots overlapped in the scatter plot with a 70% threshold. Therefore, to examine the false positives and negatives of visual acuity loss detection under the two conditions, a histogram of Landolt chart visual acuity at the rate of grating visual acuity was drawn (Figure 7). At a cutoff value of 70%, the low uncorrected visual acuity and severe refractive error tended to be calculated higher than the 80 cm Landolt visual acuity. At approximately 90%, participants with low uncorrected visual acuity were often classified as having a grating visual acuity rating of one or zero. Thus, the visual acuity was assumed to decline, or refractive error was more likely to be detected in a cutoff value of 90% than 70%. Therefore, the cutoff value was set to 90%.

## Discussion

To achieve automatic grating acuity measurement, this study compared the logMAR-corrected visual acuity and refractive error with visual acuity measured using an eye-tracking system in healthy adults. A correlation was found between objective refraction and the grating acuity level and between the 80 cm Landolt visual acuity and the grating acuity level, indicating that the EMR ACTUS calibration-free eye-tracking system can be used for vision screening.

In this study, a robust correlation emerged between grating acuity levels measured with the EMR ACTUS system and the visual acuity measured with the Landolt chart commonly used in Japan, particularly at 70% and 90% thresholds. Considering the scatter plots and false-negative rates discussed earlier, a cutoff value of 90% appears more suitable than 70% for achieving visual acuity measurements comparable with conventional methods. To simulate the daily clinical practice of TACs, the screen was divided into left and right areas at the center. In a two-alternative forced-choice task such as the TAC, a cutoff of 75% may be reasonable as the mean of the chance level (50%) and perfectly correct response (100%). On the other hand, our system continuously measured the gaze and estimated the time that the eye gaze stayed on either area. Therefore, a different cutoff may be optimum. The system can be configured to limit the area of interest to where the grating pattern is present. However, this setting was not used because the CALFREE mode has lower accuracy values than the standard calibration. In clinical settings, examiners typically ascertain only the direction of eye movements during TAC-based vision screening so that this setting can reproduce clinical conditions.

Jones et al. administered a binocular vision test to infants aged two to 12 months using an adaptive computerized vision test for infants employing eye tracking (ACTIVE) and Keeler infant acuity cards [15]. The performance (testability, reliability, and accuracy) of the ACTIVE system was comparable to that of the current clinical gold standard technique (acuity cards). Hathibelagal et al. conducted an objective vision test using an eye-tracking system in 20 infants aged three to 11 months and 15 adults aged 22-47 years [14]. In that study, the binocular grating acuity obtained using a conventional grating acuity test was comparable to that measured by displaying grating patterns on a screen, with a difference of <0.5 octaves. Wen et al. demonstrated that the performance (testability, reliability, and accuracy) of an automated acuity card procedure using an eye-tracking system was comparable to that of TACs [18]. These results suggest the possibility of automating grating card testing. However, the application of these automation techniques in infants, mainly using monocular vision, has not been assessed thoroughly. Studies on infants typically focus on measuring binocular vision. Thus, evaluating these techniques based on monocular vision may be essential, particularly in clinical ophthalmic practice.

Recent studies have explored new applications of eye-tracking systems in clinical ophthalmic practice, leveraging their advantage of not requiring subjective responses. Notable examples include the work of Iwata et al. who successfully automated the Hess screen test, which is used to assess the function and coordination of the extraocular muscles to aid in the diagnosis of ocular motility disorders [21]. Automating various vision screening processes will contribute to the development of a medical examination-friendly vision screening method. After the medical examination, visual acuity measurements must be easy for the ophthalmologist at the medical facility to interpret. In ophthalmology, monocular visual acuity is evaluated; thus, even at the screening stage, the measured values should be as close as possible to those obtained in ophthalmology. In this study, the developed device makes it possible to measure monocular visual acuity simply using the conventional method of occluding the non-measured eye with a hand or an occluder.

The significant technological progress in digital devices, such as smartphones and tablets, is expected to further modernize eye-tracking systems for ophthalmic use. For instance, an iPad was used for grating acuity testing in infants [22]. If eye tracking technology improves, in the future, in-home inspections may be performed using methods such as combining a TV screen and a portable eye tracker. Thus, this study will help in developing an essential technology for remote screening of amblyopia, which, if realized, will contribute to the early detection of amblyopia and the reduction of regional disparities. In addition, the visual acuity of a large sample of infants was collected by TACs in a previous study [23]. In large-scale vision surveys among pediatric populations for epidemiological studies examining visual acuity, an automated vision screening system is also anticipated to replace conventional tools such as TACs.

In Japan, under the Maternal and Child Health Act, eye examinations are part of the health checkup for three-year-old children [24]. This initiative primarily assesses visual acuity, and the primary test ensures that the visual acuities of the left and right eyes are +0.3 logMAR or higher, as measured by the parents. Parents also assess whether their children find it challenging to follow a visual target by the eyes. The secondary test is conducted at the municipal public health center, where results from the first test are collected. Children with visual acuity below +0.3 logMAR in one eye during a questionnaire survey for parents are reexamined by a public health nurse or examined by a doctor. If amblyopia or eye disease is suspected after the examination, a visit to a medical institution is recommended. Hayashi et al. proposed introducing the Spot™ Vision Screener (Welch Allyn, Inc., NY, USA) to the primary and secondary tests, which rely on questionnaires and subjective visual acuity evaluations [25]. The effectiveness of this device was reported in a cohort study [26]. In a recent survey in Japan, approximately 70% of local governments have adopted refraction testing [27]. In addition, the automatic visual acuity screening device developed in this study may eliminate subjectivity, providing a more reliable and direct assessment of amblyopia. Furthermore, the TAC technique may be effective if applied to children aged <3 years.

The EMR ACTUS eye-tracking system used in this study does not require eye gaze calibration and facilitates easy monocular vision screening. Consequently, the accuracy and false-negative rates of vision testing were investigated with the EMR ACTUS system using data from the left eyes of healthy adults. No unique system settings were necessary for monocular testing because the calibration for each eye was completed during a demonstration image displayed on the screen after the examinee sat in front of the system and was instructed to gaze at a grating pattern. Similar to conventional visual testing, a monocular grating acuity test could be initiated by covering the other eye. To the best of our knowledge, few studies have addressed calibration difficulties on the automation of grating card testing using eye tracking techniques. However, complex calibration procedures may reduce the success rate of vision screening and impede the collection of a large amount of data, particularly in infants. Calibration typically requires examinees to fixate on several visual targets displayed on a screen for at least 15 seconds before eye tracking. Infants may experience difficulty fixing their eyes on a target until calibration is completed. Although some systems offer an infant calibration mode that induces fixation on a target during calibration using operant conditioning, it does not resolve time-related issues. The system used appeared sufficient to measure the time an examinee spends focusing on grating patterns and to count the frequency that the examinees fixate their gaze on targets. This device has been used in other studies on children with severe physical and mental disabilities [28]. Considering the results of our research, this technology is anticipated to apply to automated visual acuity testing of infants.

This study has some limitations. First, it solely examined the applicability of the system for monocular vision testing in healthy adults. Our research group is currently preparing to assess its suitability for vision screening in infants and evaluate the measurement success rate, reliability, and comparability with TACs in this population. Second, the maximum spatial frequency of the grating patterns was limited to 25.8 cpd because of the display resolution, even though it was sufficient for screening. Potential solutions include employing a higher-resolution display, using a tracking system with an external display, or conducting vision testing at a longer distance (>80 cm). Finally, this study suggested that visual acuity can be measured using EMR ACTUS by instructing adults to look at the grating patterns. However, conventional TACs utilize preferential looking, one of the infants' instinctive behaviors. It remains to be studied whether instinctive behavior can be evoked in infants using EMR ACTUS, like TACs. Instinctive behavior may not occur on a monitor screen. These subjects need to be evaluated in further research.

## Conclusions

This study demonstrated the feasibility of monocular grating acuity testing and simulating TAC testing in healthy adults using a newly developed calibration-free eye-tracking system. Under the current system, the visual acuity cutoff was validated to ensure a correlation between the system-derived grating acuity and the Landolt visual acuity. The results suggest that a target might be recognized if examinees have fixated their eyes on the area with a grating pattern 90% of the measurement time. Establishing a vision screening technique using the EMR ACTUS system requires collecting monocular vision data from infants and verifying the correlation with visual acuity obtained using a conventional method.
